# Fabrication of CeO_2_–MO*_x_* (M = Cu, Co, Ni) composite yolk–shell nanospheres with enhanced catalytic properties for CO oxidation

**DOI:** 10.3762/bjnano.8.241

**Published:** 2017-11-16

**Authors:** Ling Liu, Jingjing Shi, Hongxia Cao, Ruiyu Wang, Ziwu Liu

**Affiliations:** 1 Low Carbon Energy Institute, China University of Mining and Technology, Xuzhou 221008, People’s Republic of China; 2 Key Laboratory of Coal-Based CO2 Capture and Geological Storage of Jiangsu Province, China University of Mining and Technology, Xuzhou 221008, People’s Republic of China; 3 School of Chemical Engineering and Technology, China University of Mining and Technology, Xuzhou 221116, People’s Republic of China

**Keywords:** CeO_2_, CO oxidation, surface decoration, synergistic interaction, yolk–shell structure

## Abstract

CeO_2_–MO*_x_* (M = Cu, Co, Ni) composite yolk–shell nanospheres with uniform size were fabricated by a general wet-chemical approach. It involved a non-equilibrium heat-treatment of Ce coordination polymer colloidal spheres (Ce-CPCSs) with a proper heating rate to produce CeO_2_ yolk–shell nanospheres, followed by a solvothermal treatment of as-synthesized CeO_2_ with M(CH_3_COO)_2_ in ethanol solution. During the solvothermal process, highly dispersed MO*_x_* species were decorated on the surface of CeO_2_ yolk–shell nanospheres to form CeO_2_–MO*_x_* composites. As a CO oxidation catalyst, the CeO_2_–MO*_x_* composite yolk–shell nanospheres showed strikingly higher catalytic activity than naked CeO_2_ due to the strong synergistic interaction at the interface sites between MO*_x_* and CeO_2_. Cycling tests demonstrate the good cycle stability of these yolk–shell nanospheres. The initial concentration of M(CH_3_COO)_2_·*x*H_2_O in the synthesis process played a significant role in catalytic performance for CO oxidation. Impressively, complete CO conversion as reached at a relatively low temperature of 145 °C over the CeO_2_–CuO*_x_*-2 sample. Furthermore, the CeO_2_–CuO*_x_* catalyst is more active than the CeO_2_–CoO*_x_* and CeO_2_–NiO catalysts, indicating that the catalytic activity is correlates with the metal oxide. Additionally, this versatile synthesis approach can be expected to create other ceria-based composite oxide systems with various structures for a broad range of technical applications.

## Introduction

As one of the most important rare-earth oxides, ceria (CeO_2_) has attracted a great deal of research attention due to its high oxygen storage capacity (OSC) and good redox properties [1–3]. Because of these unique characteristics, CeO_2_ has been widely used as environmental catalysts for the removal of harmful pollutants from exhaust gases, such as oxidation of low-concentration of CO [4], selective reduction of NO*_x_* with NH_3_ [5], and oxidation of volatile organic compounds (VOC) [6–7]. The catalytic activity of CeO_2_ is believed to originate from the reversible transformation between Ce^4+^ and Ce^3+^ and affected by various structural factors [8–10], including high surface area, preferential exposure of reactive facets and oxygen vacancy defects. However, pure CeO_2_ deactivates seriously at an elevated temperature due to the decline of surface area and OSC. In order to maintain the OSC and enhance the catalytic activity and thermal stability of CeO_2_, CeO_2_-based composite oxides by combining ceria with other low-valence metal oxides have been widely studied. Importantly, due to the synergistic effect between the two components, CeO_2_-based composite oxides exhibit a remarkable catalytic activity that is comparable with or even superior to that of noble metal-based catalysts in some catalytic reactions [11–12].

So far, a remarkable process has been developed for the synthesis of CeO_2_-based composite oxides, including CeO_2_–CuO*_x_* [13], CeO_2_–ZnCo_2_O_4_ [14], CeO_2_–CoO*_x_* [15], CeO_2_–MnO*_x_* [16], CeO_2_–ZnO [17], CeO_2_–Fe_2_O_3_ [18], and CeO_2_–ZrO_2_ systems [19]. Taking the CeO_2_–CuO catalyst as a typical example, the improved catalytic activity is closely related to the synergistic interaction between copper and ceria, which promotes the exchange of charges between Ce^4+^/Ce^3+^ and Cu^2+^/Cu^+^ and leads to faster oxidation and reduction than that of the corresponding independent forms. The formation of highly-dispersed copper species promotes the adsorption of CO molecules, while the presence of oxygen vacancies provided by CeO_2_ can in turn create active oxygen in the oxidation reactions [12–13]. Therefore, the creation of two-phase interfaces as numerous as possible and, thus, the facilitation of synergistic interaction between two components are necessary to optimize the catalytic performances. The unique structure and texture of CeO_2_-based catalysts is also associated with high activity and stability in the catalytic reaction. For instance, rod-like CeO_2_–CuO catalysts with highly dispersed copper oxide clusters as active species had been reported to exhibit superior activity toward CO oxidation in contrast with commonly used CeO_2_/CuO composite catalysts [13]. Consequently, the construction of ceria-based composite oxides with pore features, hollow structure or/and hierarchical architecture, which possess excellent redox properties and abundant oxygen vacancies, will be favorable for the enhancement of catalytic activity toward CO oxidation.

CeO_2_-based hybrid oxides with hollow structure can be synthesized by a sacrificial-template method based on interfacial oxidation–reduction under mild conditions. For example, Mn_3_O_4_/CeO_2_ hybrid nanotubes were created by a template-based process involving a redox reaction between the cryptomelane-type manganese oxide nanowire template and Ce(NO_3_)_3_ [20]. Ce–Mn nanotubes were also fabricated by treating Ce(OH)CO_3_ templates with aqueous KMnO_4_ solution and subsequent selective washing with HNO_3_ to remove the residual Ce(OH)CO_3_ [21]. In another case, well-dispersed MnO_2_@CeO_2_–MnO_2_ and CeO_2_–CuO*_x_* composite hollow spheres were synthesized through a facile reflux method using carbon spheres as sacrificial templates. The obtained material showed a high catalytic activity for CO oxidation [16,22]. In addition, porous/hollow CeO_2_-based composite oxides with high surface area can be prepared through heat treatment of suitable cerium-containing precursors. Typically, uniform porous Ce_1−_*_x_*Zn*_x_*O_2−δ_ solid-solution nanodisks were synthesized by thermal decomposition of the as-prepared Ce–Zn precursor and exhibited excellent activity for removing CO [17]. CeO_2_–ZnO composite hollow microspheres were fabricated via annealing of a precursor of amorphous zinc–cerium citrate hollow microspheres and presented excellent catalytic activity in CO oxidation [23]. Porous CeO_2_:Cu^2+^ materials with a tunable surface area were prepared through the thermolysis of a nanosized CeCu(BTC)(H_2_O)_6_ precursor [24]. Impressively, Ce_2_(SO_4_)_3_ was employed as the precursor to synthesize mesoporous CeO_2_–CuO bimetal oxide nanorods without the need for additional heat treatment, and the resultant sample exhibited enhanced catalytic activity in the oxidation of CO [25]. Otherwise, as a special composite structure, heterogeneous core@shell structures are believed to integrate the function of individual nanocrystals and induce the unique synergetic catalytic activities. For instance, ZnCo_2_O_4_@CeO_2_ core@shell spheres [14] and Co_3_O_4_@CeO_2_ core@shell cubes [26] with tunable CeO_2_ shell thickness were prepared by a facile self-assembly method and exhibited promising performance in the catalytic oxidation of CO. Consequently, a suitable choice of templates or cerium-containing precursors and the control of the various factors that govern the morphology, texture and physico-chemical properties, provide a promising approach to fabricate CeO_2_-based mixed oxide with various nanostructures. However, compared with pure CeO_2_, it remains a challenge to synthesize CeO_2_-based mixed oxides with well-defined morphology, tunable chemical composition and distribution, and desirable physico-chemical properties.

Herein, we report a general approach to fabricate uniform CeO_2_–MO*_x_* (M = Cu, Co, Ni) composite yolk–shell nanospheres with highly dispersed MO*_x_* species, in which CeO_2_ yolk–shell nanospheres were first constructed by non-equilibrium heat treatment of a Ce-CPCSs precursor and subsequent treatment with M(CH_3_COO)_2_ in a solvothermal process. Due to the well dispersion of MO*_x_* and the close contact between CeO_2_ and MO*_x_*, the resultant nanospheres exhibited improved catalytic activity in the oxidation of CO.

## Results and Discussion

### Formation and characterization

**Figure 1:**
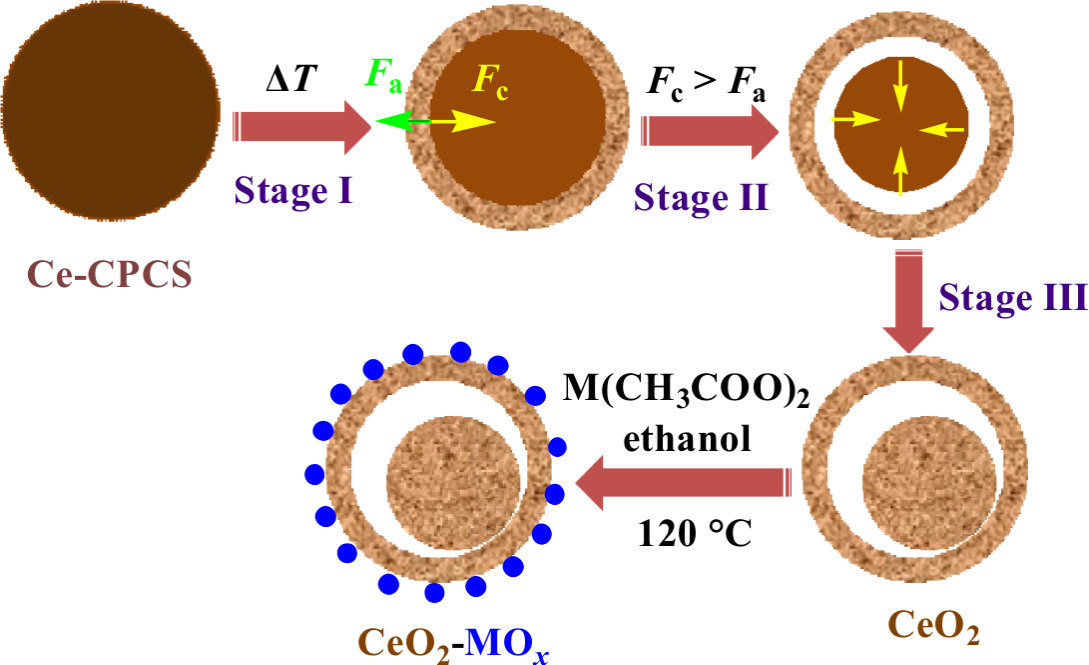
Schematic illustration of the formation of CeO_2_–MO*_x_* composite yolk–shell nanospheres.

The synthetic mechanism for the CeO_2_–MO*_x_* yolk–shell nanospheres is illustrated in Figure 1. First, according to the reported method [27], well-dispersed Ce-CPCSs with uniform size were synthesized on the basis of the integration of coordination chemistry with anti-solvent effects for synchronized precipitation. Second, the resultant Ce-CPCSs served as the precursor to produce CeO_2_ yolk–shell nanospheres by non-equilibrium heat treatment with a proper heating rate. Heterogeneous contraction, caused by non-equilibrium heat treatment process, is regarded as a promising and effective approach to controllably design hollow structures with single and multilevel shells. The complexity of the shell structures is generally determined by the difference between the cohesive force (*F*_c_) and the adhesive force (*F*_a_) created by a proper temperature ramping rate [28–30]. In the early stages of heat treatment, at a high heating rate, a temperature gradient (Δ*T*) along the radial direction leads to the generation of a dense CeO_2_ shell at the surface of the Ce-CPCS core (stage I). Then, heterogeneous contraction takes place because the balance between the opposite forces *F*_c_ and *F*_a_ is disturbed. When *F*_c_ > *F*_a_ at a high Δ*T*, the inner core contracts inward and detaches from the preformed outer shell (stage II). During further annealing, the inner core shrinks into a solid structure and consequently CeO_2_ yolk–shell spheres form (stage III). Upon solvothermal treatment, M(CH_3_COO)_2_ is hydrolyzed to M(OH)_2_ and subsequently transformed into MO*_x_* and finally deposited onto the surface of CeO_2_ yolk–shell spheres, leading to the formation of CeO_2_−MO*_x_* composite yolk–shell spheres.

**Figure 2:**
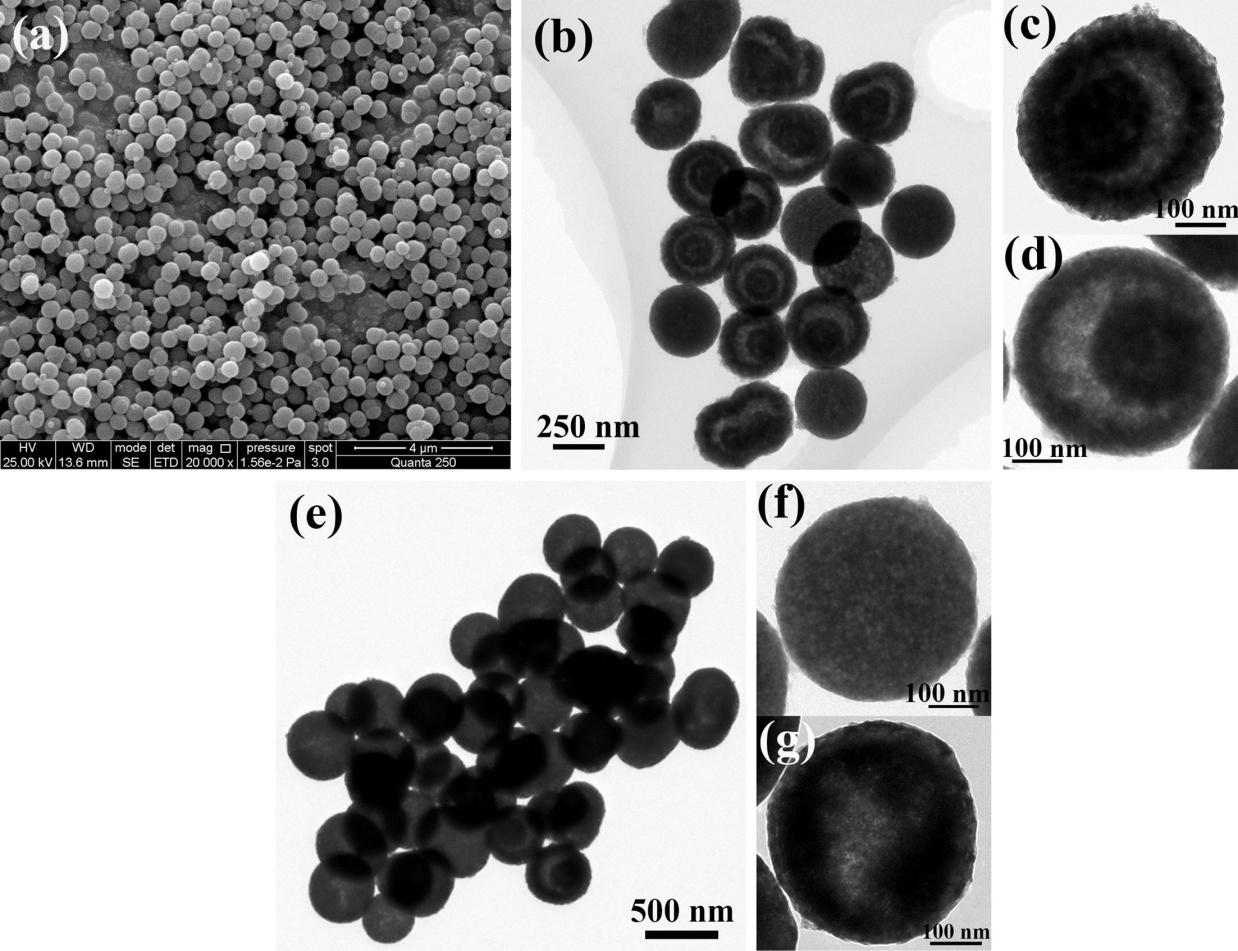
SEM image of Ce-CPCSs (a) and TEM images of (b–d) the CeO_2_ yolk-shell nanospheres (heating rate 8 °C/min) and of (e–g) solid nanospheres (heating rate 2 °C/min).

The morphology of the obtained samples was investigated by using scanning electron microscopy (SEM) and transmission electron microscopy (TEM). The as-synthesized Ce-CPCSs precursor exhibits a well-dispersed solid spherical structure in the range of 400–500 nm (Figure 2a). It has been reported that, during the heating process, the calcination temperature and time is correlated with the crystalline structure of the as-obtained oxides, while a proper heating rate is necessary for the controllable synthesis of hollow structures with different multilevel interiors [30]. In our present work, when the Ce-CPCSs precursor was calcined at 500 °C with a heating rate of 8 °C/min, most of the obtained CeO_2_ nanostructures present a yolk–shell spherical morphology with a shell thickness of about 50 nm (Figure 2b–d). In contrast, at a low heating rate of 2 °C/min, the Ce-CPCS spheres were almost homogeneously heated from the surface to the center and resulted in the formation of porous solid spheres along with some hollow structures (Figure 2e–g).

**Figure 3:**
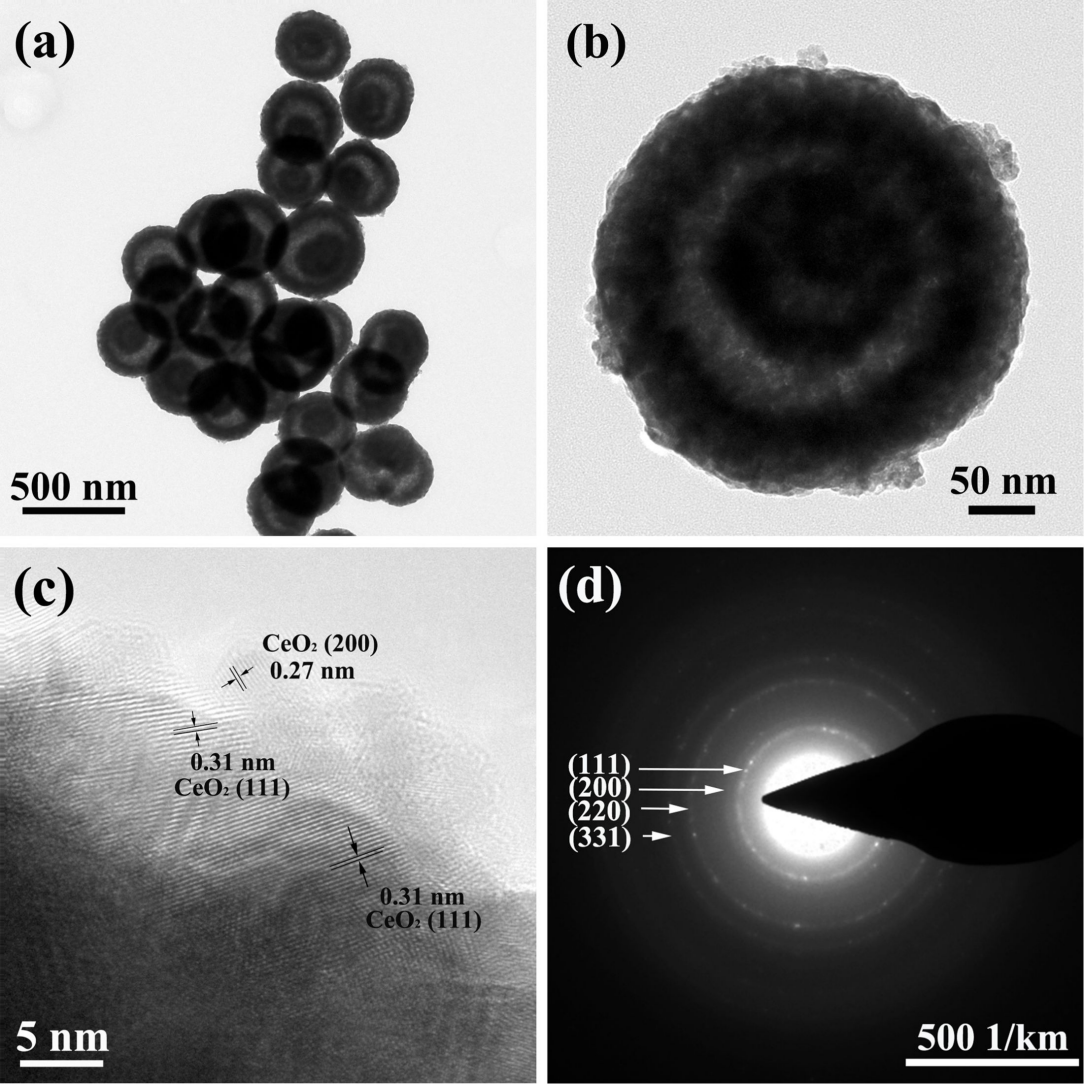
Low-magnification TEM images (a, b), high-magnification TEM image (c) and the SAED pattern (d) of the CeO_2_−CuO*_x_* composite yolk-shell nanospheres.

**Figure 4:**
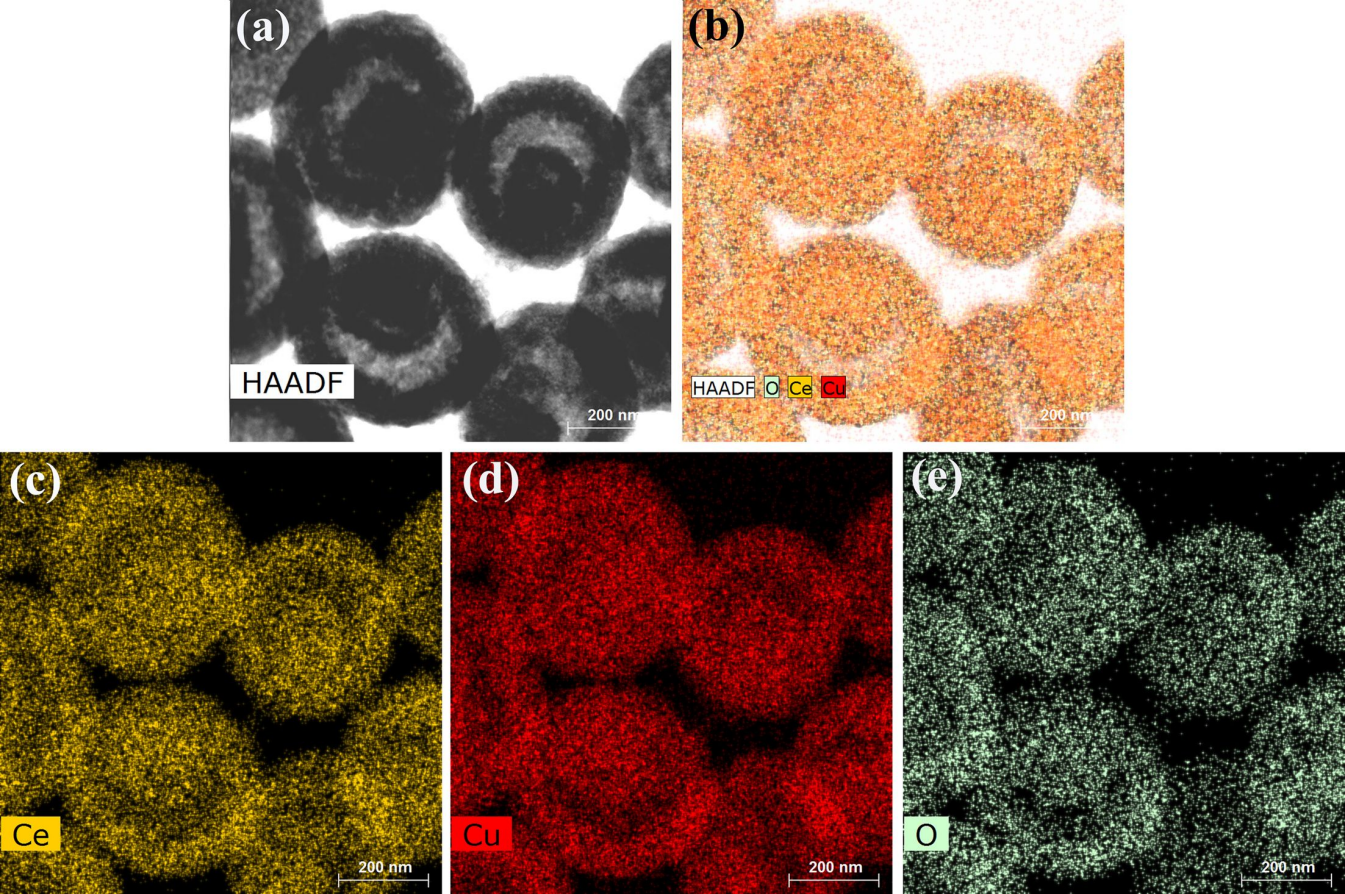
TEM image (a) and its mapping analysis (b–e) of the CeO_2_–CuO*_x_* composite yolk–shell nanospheres.

After further reaction between the yolk–shell structured CeO_2_ and Cu(CH_3_COO)_2_ at 120 °C, the resultant CeO_2_–CuO*_x_* composites still maintained the yolk–shell morphology without any obvious change after the harsh solvothermal treatment. The morphology of the CeO_2_–CuO*_x_* yolk–shell spheres can be observed from TEM images in Figure 3. The strong contrast between outer shell and inside cavity indicates the hollow structure of the spheres. Besides, the size of CeO_2_–CuO*_x_* composite yolk–shell nanospheres ranges from 400 to 500 nm with a shell thickness of approximately 50 nm (Figure 3b). The high-resolution TEM image is displayed in Figure 3c. The lattice fringe spacing of 0.31 nm and 0.27 nm can be indexed to the (111) and (200) crystal planes of face-centered-cubic fluorite-type CeO_2_, respectively. No lattice spacing can be corresponded to Cu species. In the corresponding selected-area electron diffraction (SAED) pattern in Figure 3d only the diffraction rings belonging to CeO_2_, indicative of a polycrystalline structure, can be recognized. This result is similar to the previously reported literature [22]. Energy-dispersive X-ray (EDX) analysis was employed to further investigate the elemental distribution of Ce and Cu species in the yolk–shell nanospheres. In Figure 4, the elements Ce, Cu, and O are represented by yellow, red, and green colors, respectively. A uniform distribution of Ce, Cu, and O in each yolk–shell sphere, which is favorable for the synergistic interaction between CeO_2_ and CuO*_x_,* can clearly be seen.

**Figure 5:**
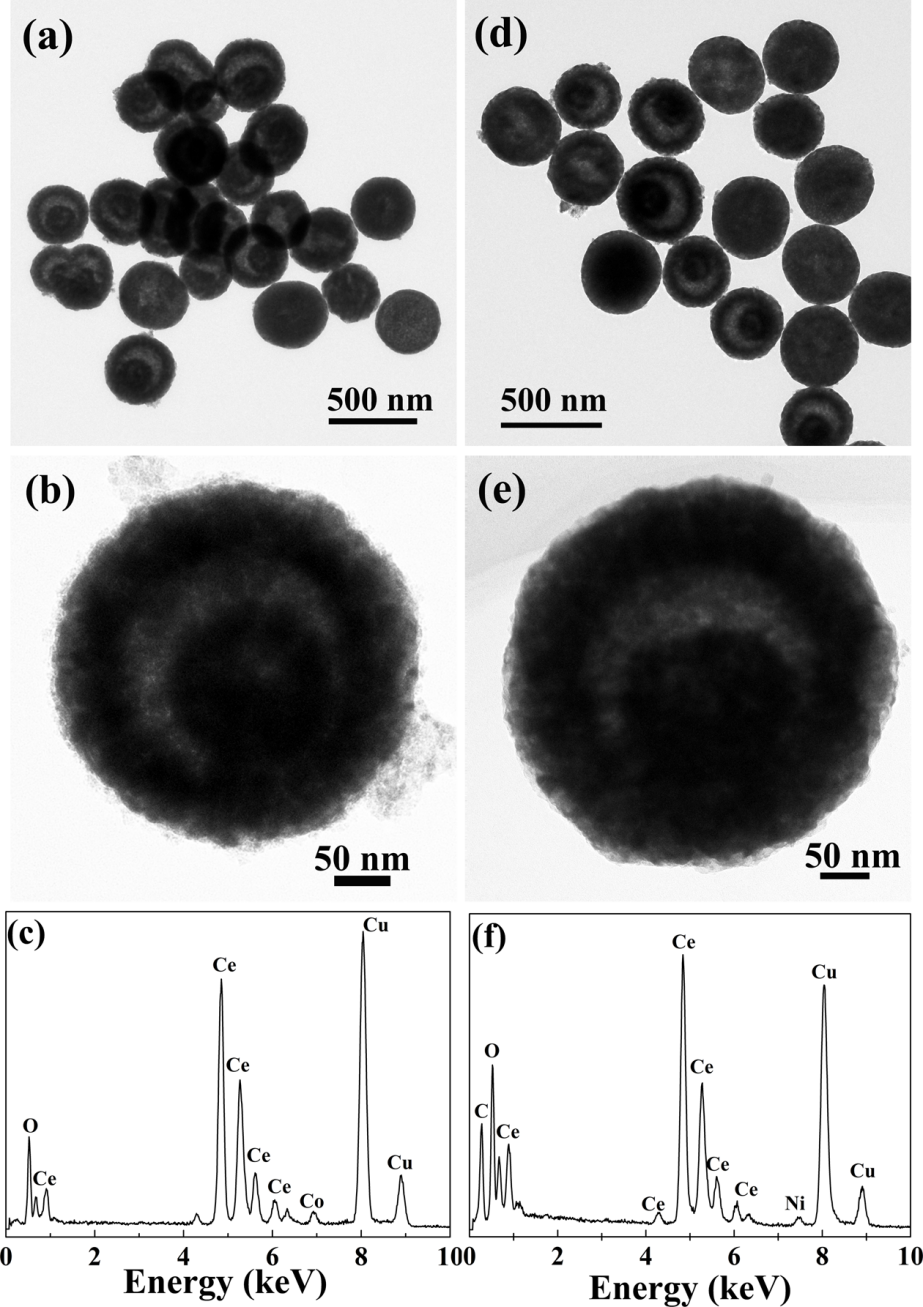
TEM images and the corresponding EDX patterns of the synthesized CeO_2_–CoO*_x_* (a–c) and CeO_2_–NiO (d–f) composite yolk–shell nanospheres.

Ce–Co and Ce–Ni composite oxide nanostructures were prepared by a similar solvothermal process in which CeO_2_ yolk–shell nanospheres were mixed with Co(CH_3_COO)_2_ or Ni(CH_3_COO)_2_ in ethanol solution. Spherical yolk–shell structures in the range of 400–500 nm are clearly observed in the TEM images (Figure 5a,b,d,e). The coexistence of Ce and Co or Ni is further confirmed by the EDX spectra (Figure 5c,f). These results indicate the versatility of this synthesis approach in the preparation of CeO_2_-based transition-metal mixed-oxide nanostructures. Based on this synthesis method, other CeO_2_-based composite oxides with various nanostructures can be expected to be fabricated through the pre-formation of CeO_2_ or CeO_2_-based solid solution with different morphologies and the subsequent decoration of highly dispersed transition-metal oxide cluster species.

**Figure 6:**
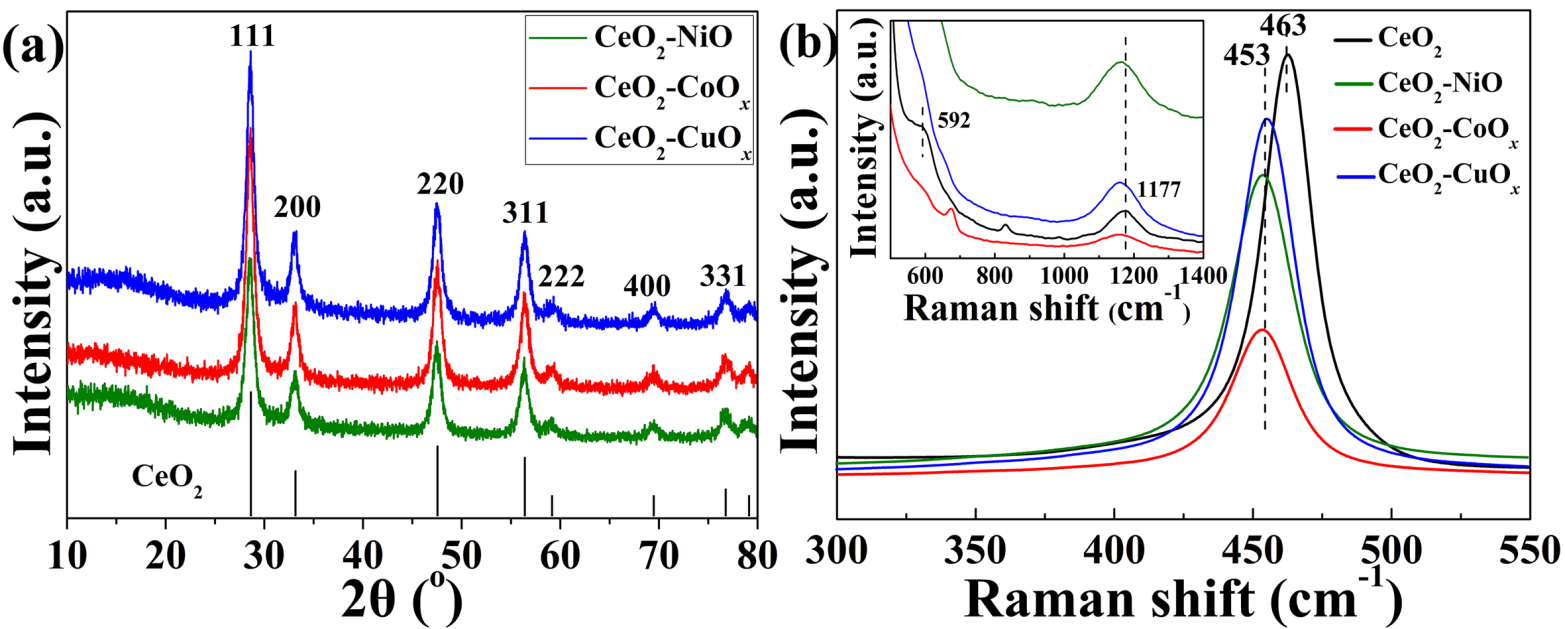
XRD patterns (a) and Raman spectra (b) of the as-synthesized CeO_2_–MO*_x_* composite yolk–shell nanospheres.

Information about crystallinity and phases of the samples were obtained from X-ray diffraction (XRD) analysis. Figure 6a displays the XRD patterns of the as-synthesized CeO_2_–MO*_x_* nanospheres. All diffraction peaks can be assigned to the fluorite-like cubic phase of CeO_2_ (JCPDS no. 34-0394). No diffraction peaks can be indexed to the MO*_x_* structure. The results indicate the high dispersion of MO*_x_* species onto the surface of CeO_2_ or/and the introduction of M cations into the CeO_2_ lattice.

The Raman spectra of the CeO_2_–MO*_x_* yolk–shell spheres are illustrated in Figure 6b. All of the spectra reveal a main band at 450–465 cm^−1^, which can be assigned to the F_2g_ mode of the fluorite structure of CeO_2_. Compared to pure CeO_2_, the peak intensity of the CeO_2_–MO*_x_* composite spheres decreases and the peak position shifts from 463 to 453 cm^−1^. The peak shift depends on various parameters, including the crystal defects, oxygen vacancies, phonon confinement, and inhomogeneous strain related to the reduced ceria [31–32]. Herein, the shift can be related to the interaction between the MO*_x_* species and CeO_2_ surface, which leads to lengthening and weakening of the M–O bond by sharing oxygen at the interface [29–30]. In addition, the incorporation of dopants can also result in shifts of the peak positions. This is, e.g., because the ion radius of M^2+^ (Cu^2+^ = 0.72 Å, Co^2+^ = 0.79 Å, Ni^2+^ = 0.83 Å) is much smaller than that of Ce^4+^ (1.01 Å). Also, when a small number of M^2+^ is embedded into the CeO_2_ lattice and replaces Ce^4+^, the Raman band shifts to lower wavenumbers because additional oxygen vacancies form to compensate for the valence mismatch between M^2+^ and Ce^4+^ ions [31]. Additionally, the weak peaks of pure CeO_2_ at 592 and 1177 cm^−1^ can be assigned to the D (defect-induced mode) and 2LO (second-order longitudinal optical mode) bands, respectively, which indicate the amount of surface oxygen vacancies [17]. The bands become more pronounced and shifts to lower wavenumbers for CeO_2_–MO*_x_* samples. It appears that CeO_2_–MO*_x_* samples, especially CeO_2_–CuO*_x_* and CeO_2_–NiO, generate more surface oxygen vacancies than pure CeO_2_.

**Figure 7:**
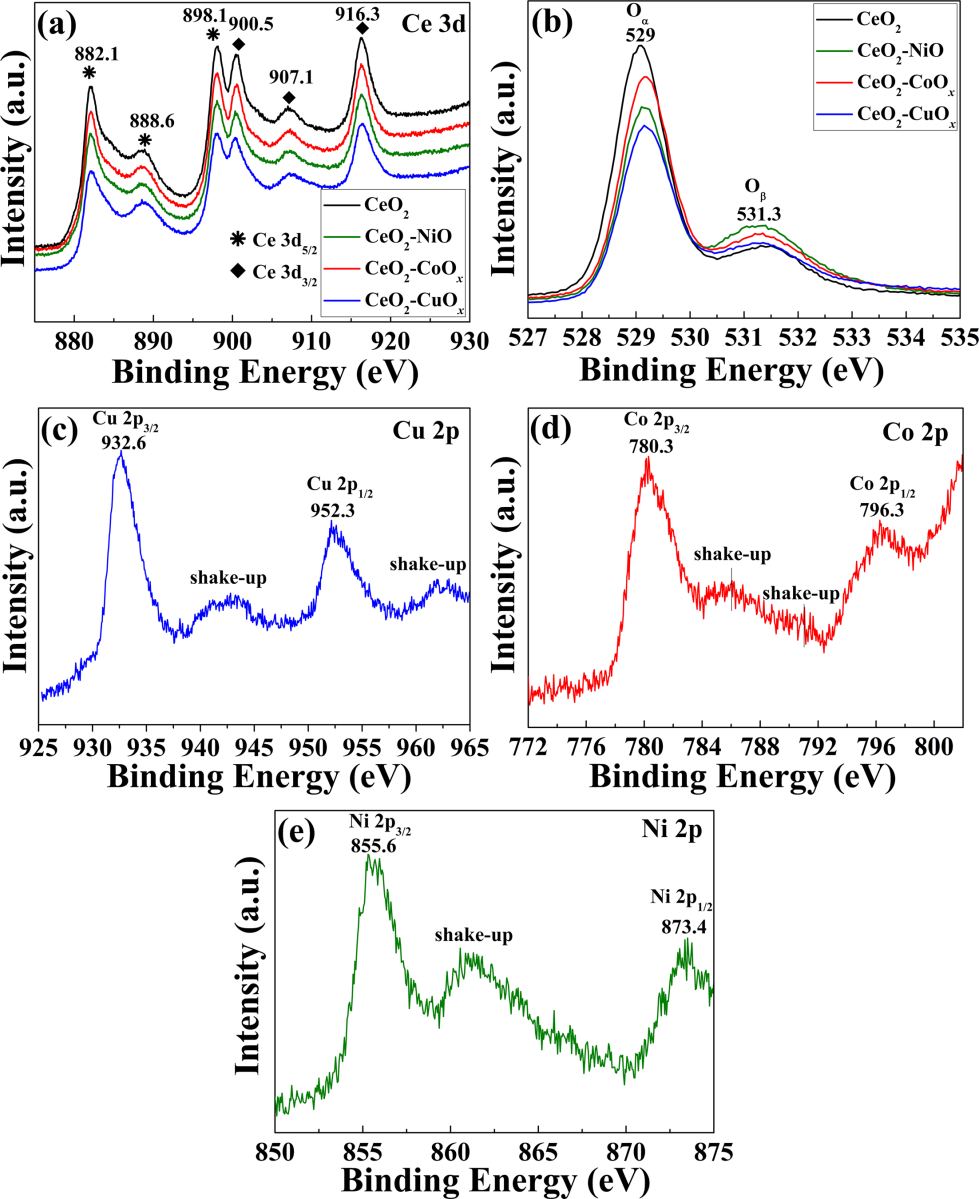
XPS spectra of Ce 3d (a), O 1s (b), Cu 2p (c), Co 2p (d) and Ni 2p (e) of the as-synthesized CeO_2_–MO*_x_* composite yolk–shell nanospheres.

In order to obtain detailed information about the chemical bonding states of the as-prepared CeO_2_–MO*_x_* yolk-shell nanospheres, X-ray photoelectron spectroscopy (XPS) analyses were carried out and the results are shown in Figure 7. As shown in Figure 7a, all samples exhibit similar profiles in the Ce 3d spectral region, which clearly represents a typical Ce(IV) 3d spectrum. Peaks centered at 882.1, 888.6, and 898.1 eV can be assigned to Ce(IV) 3d_5/2_, while peaks located at 900.5, 907.1, and 916.3 eV can be indexed to Ce(IV) 3d_3/2_, respectively [12,20]. In the O 1s spectra in Figure 7b, two peaks marked as O_α_ and O_β_ are clearly identified. According to the literature, the main peak O_α_ at a binding energy of 529 eV is corresponding to lattice oxygen, while the shoulder peak O_β_ at a binding energy of 531.3 eV is ascribed to defective or adsorptive oxygen species, such as low-coordinated oxygen atoms, chemisorbed oxygen, or hydroxy groups [12]. It is well-accepted that O_β_ species are more active than O_α_ species due to their higher mobility. With the stronger O_β_ peak, the CeO_2_–MO*_x_* samples are expected to be richer in surface-active oxygen and have a better capacity for oxygen storage. In the Cu 2p spectrum (Figure 7c), two pronounced peaks at about 932.6 and 952.3 eV can be attributed to Cu 2p_3/2_ and Cu 2p_1/2_ of Cu(I), respectively, whereas a broad shake-up peak observed at around 946 eV can be assigned to the presence of Cu(II) [33]. It is therefore clear that the CeO_2_–CuO*_x_* sample contains coexisting Cu^+^/Cu^2+^ oxidation states. In the Co 2p spectrum (Figure 7d), two strong peaks at 780.3 eV assigned to Co 2p_3/2_ and 796.3 eV to Co 2p_1/2_, along with two shake-up peaks at around 785.6 and 791 eV, are observed, indicating the coexistence of Co(II) and Co(III) in the CeO_2_–CoO*_x_* sample. It has been reported that the oxidation state of Co can be distinguished by the binding energy and the intensity of shake-up satellites of the Co 2p photo-peak. Generally, Co^2+^ shows two obvious shake-up satellites at around 785 and 802 eV, while pure Co^3+^ displays only a very weak shake-up peak at about 791 eV. If Co^2+^/Co^3+^ oxidation states coexist, a plateau in the range of 783–792 eV will be observed instead of two distinct shake-up peaks [31,34]. In Ni 2p spectrum of the CeO_2_–NiO sample (Figure 7e), the binding energies at 855.6 and 873.4 eV are ascribed to the characteristics Ni 2p_3/2_ and Ni 2p_1/2_ signals of Ni(II), while the peak at around 861.1 eV is the shake-up peak at the high-energy side of the Ni 2p_3/2_ edge [31].

Additionally, direct quantification of surface concentration ratio of M has been estimated by the integrated intensities of the M 2p and Ce 3d XPS peaks. As shown in Table 1, the surface M/(M + Ce) ratio calculated from XPS data is 37%, 22% and 28% for CeO_2_–CuO*_x_*, CeO_2_–CoO*_x_* and CeO_2_–NiO, respectively. Compared with 11.3%, 8.3% and 6.6% for the actual concentration measured by inductively coupled plasma mass spectrometry (ICP-MS) analysis, the surface concentration is higher than the actual concentration, which indicates the surface enrichment of MO*_x_* species. The high concentration of MO*_x_* species on the CeO_2_ surface may result in the enhanced catalytic activity of the as-synthesized CeO_2_–MO*_x_* samples.

**Table 1:** Chemical composition of the CeO_2_–MO*_x_* composite yolk–shell nanospheres calculated from ICP-MS and XPS analyses.

samples	CeO_2_–CuO	CeO_2_–CoO*_x_*	CeO_2_–NiO

actual M/(M + Ce) ratio (atom %)^a^	11.3	8.3	6.6
surface M/(M + Ce) ratio (atom %)^b^	37	22	28

^a^From ICP-MS analysis; ^b^from XPS analysis.

**Figure 8:**
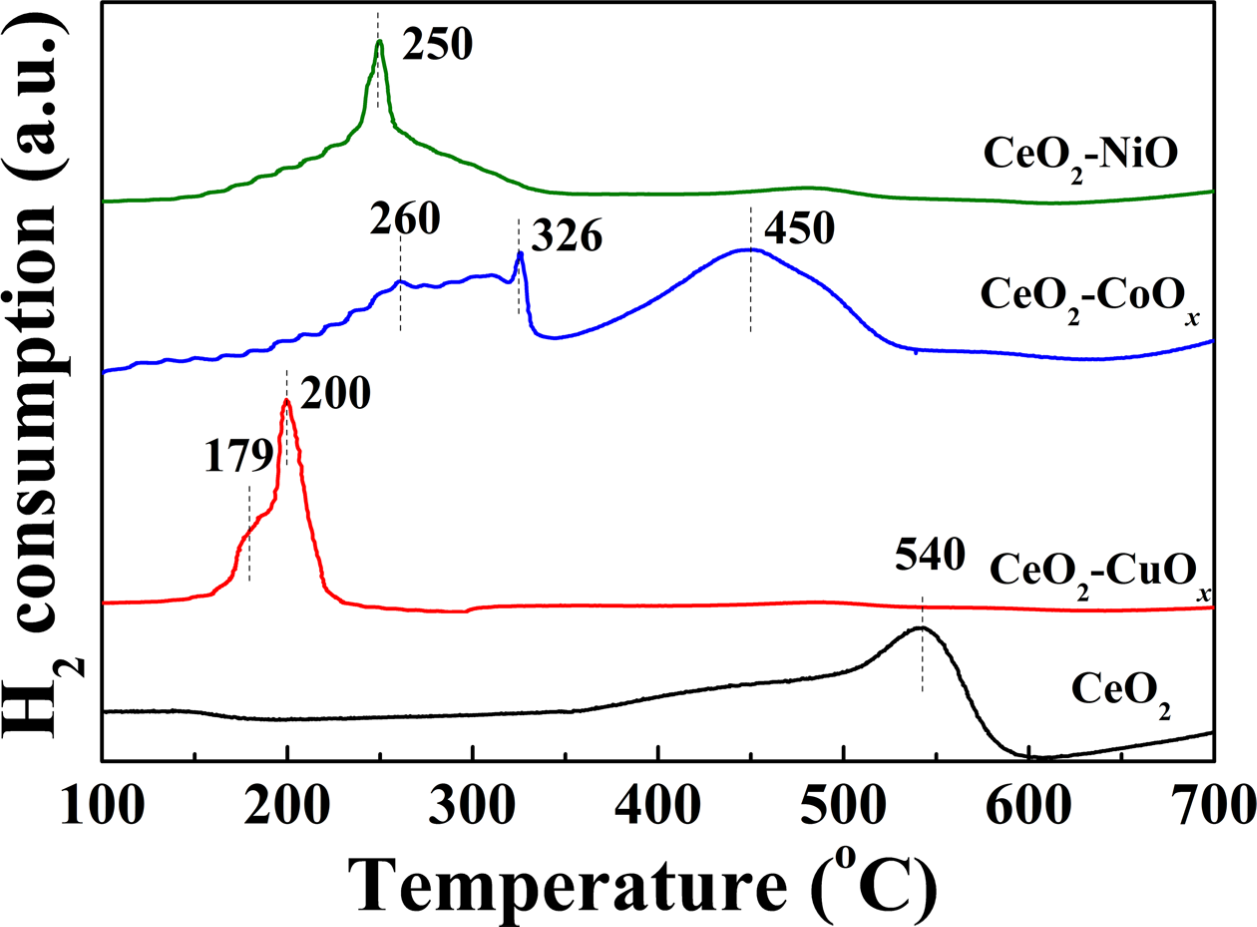
H_2_-TPR profiles of as-prepared CeO_2_, CeO_2_–CuO*_x_*, CeO_2_–CoO*_x_* and CeO_2_–NiO yolk–shell nanospheres.

In order to further understand the effect of the interaction between CeO_2_ and MO*_x_* on the redox properties of these binary oxides, hydrogen temperature-programmed reduction (H_2_-TPR) analysis was performed on the various CeO_2_–MO*_x_* composite yolk–shell nanospheres. As displayed in Figure 8, pristine CeO_2_ shows a clear reduction peak at 540 °C, which is attributed to the reduction of surface-oxygen species (capping oxygen). After the introduction of the CuO*_x_* phase, the surface-reduction peak is changed significantly and new peaks appear below 300 °C. For the CeO_2_–CuO*_x_* composite yolk–shell nanospheres, two major reduction peaks are observed. The wide low-temperature band centered at 179 °C originates from the highly dispersed CuO*_x_* clusters, while the sharp high-temperature peak located at 200 °C is due to the strong interactions in the Cu–[O]–Ce structure [13]. In general, commercial Cu_2_O and pure CuO synthesized using a conventional precipitation method exhibit a H_2_ consumption peak in the range of 240–300 °C, related to the reduction in the pure bulk CuO*_x_* phase [25,35]. For comparison, the binary Ce–Cu oxide shows relatively low reducibility compared to pure ceria and copper oxides due to the significant interaction between the two phases.

In the case of CeO_2_–CoO*_x_* sample, three reduction peaks are clearly displayed. Two weak peaks are located at relatively low temperatures of 260 and 326 °C, while the other board one is centered at 450 °C. According to the previous reports [11,36], the reduction peaks of CeO_2_–Co_3_O_4_ catalysts in the H_2_-TPR profiles can be generally divided into four temperature regions: α (< 240 °C), β (240–320 °C), γ (320–480 °C), and δ (480–700 °C). The peaks α and β are assigned to the reduction of adsorptive oxygen species on the surface and Co^3+^ at the CeO_2_–Co_3_O_4_ contact interface to Co^2+^, respectively; while the peaks γ and δ are attributed to the reduction of Co_3_O_4_ and Co^2+^ which interacted with the CeO_2_ support to metal Co, respectively. On basis of these reduction patterns, the H_2_ consumption peaks at 260 and 326 °C of the CeO_2_–CoO*_x_* sample in our present work could be ascribed to the reduction of Co^3+^ interacting with CeO_2_ at the interface to Co^2+^ and the reduction of CoO*_x_* weakly interacting with CeO_2_ directly to metal Co, respectively. The broad reduction peak at 450 °C can be related with the reduction of Co^2+^ interacting with CeO_2_ to metal Co and Ce^4+^ cations at the interface between CoO_x_ and CeO_2_. In addition, the pure Co_3_O_4_ bulk gets reduced at a high temperature of above 280 °C in the literature [6,11]. For the H_2_-TPR profile of CeO_2_–NiO sample, a sharp peak is clearly observed at around 250 °C, which can be due to the reduction of NiO cluster species weakly interacting with CeO_2_ [37]. Comparatively, Ni^2+^ ions were reduced to Ni^0^ at the temperature of 330–430°C in pure NiO sample in the literature [38]. These results reveal that the strong interaction between the MO*_x_* species and CeO_2_ is created at the two-phase interface of CeO_2_−MO*_x_* composite yolk-shell nanospheres.

### Catalytic performance

**Figure 9:**
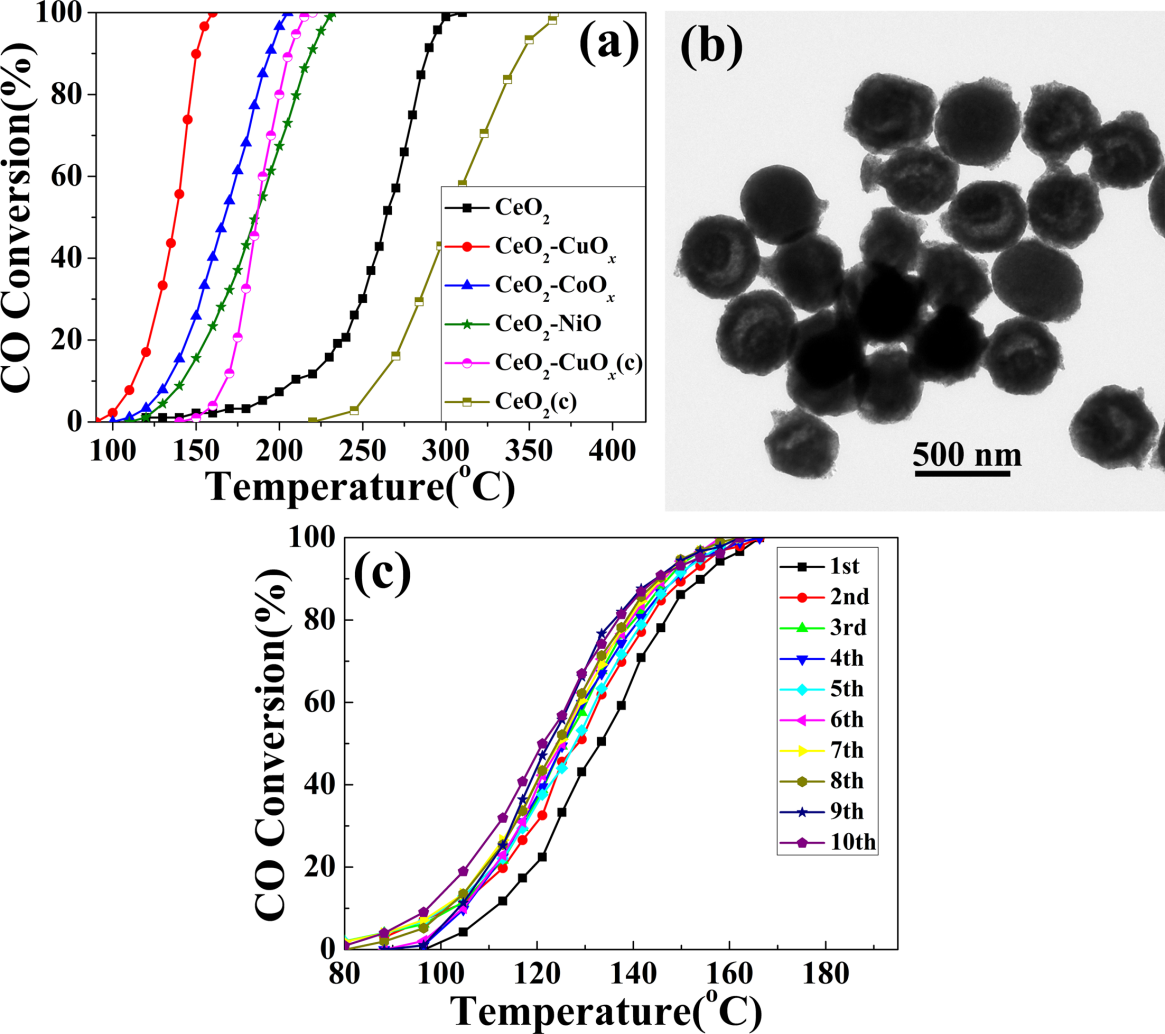
(a) CO conversions as a function of temperature for CeO_2_ and CeO_2_–MO*_x_* composite yolk–shell nanospheres, and naked and CuO*_x_*-decorated commercial CeO_2_ particles (denoted as CeO_2_(c) and CeO_2_−CuO*_x_*(c), respectively), WHSV = 60000 mL g^-1^ h^-1^; (b) TEM image of the CeO_2_–CuO*_x_* composite yolk–shell nanospheres after catalytic test; and (c) cycling test of the CeO_2_–CuO*_x_* composite yolk–shell nanospheres for CO conversion.

CO oxidation as a model reaction was carried out to evaluate the catalytic performance of the CeO_2_–MO*_x_* composite yolk–shell nanospheres and pristine CeO_2_ for comparison. Figure 9 presents the catalytic activities for the above samples. As can be seen, the temperatures for 50% CO oxidation (T_50_) of CeO_2_–CuO*_x_*, CeO_2_–CoO*_x_* and CeO_2_–NiO were 137, 167, and 185 °C, respectively, with a large temperature difference to pure CeO_2_ (206 °C). Furthermore, complete CO conversion is obtained at 310 °C in the presence of pure CeO_2_. This is in stark contrast to 160 °C with CeO_2_–CuO*_x_*, 203 °C with CeO_2_–CoO*_x_* and 235 °C with CeO_2_–NiO, respectively. Obviously, the CeO_2_–MO*_x_* composite yolk–shell spheres showed much higher catalytic activity than pure CeO_2_ yolk–shell spheres. Because the sample of solely CeO_2_ has similar structural features to those of CeO_2_–MO*_x_*, the enhanced performance can be attributed to the decoration of MO*_x_* onto the CeO_2_ surface and the strong synergistic interaction between MO*_x_* species and CeO_2_. For comparison, a commercial CeO_2_ material was measured under the same conditions for CO oxidation. Complete CO conversion was obtained at 367 and 221 °C over the naked and CuO*_x_*-decorated commercial CeO_2_ particles, respectively. By comparison, the yolk–shell nanospheres are more active than the commercial material under the same test conditions. The higher catalytic performances of our present samples could be related to the unique yolk–shell structure. During the catalytic process, CO molecules may easily diffuse into the cavities of the CeO_2_–CuO*_x_* yolk–shell structure and then contact more active interface sites, thus enhancing the CO oxidation activity. Figure 9b shows the TEM image of the Ce–Cu binary oxides yolk–shell spheres after the catalytic test. The yolk–shell nanosphere morphology is largely retained during the catalytic oxidation, suggesting an excellent structural stability. To further explore the durability of the CeO_2_–MO*_x_* catalyst, the CeO_2_–CuO*_x_* sample was employed as a typical example and a cycling test was performed. As shown in Figure 9c, the CeO_2_–CuO*_x_* sample still maintained 100% CO conversion at 165 °C after ten successive cycles. Interestingly, the catalytic activity in the 2nd to 10th run was not reduced, and even slightly higher than that in the first run. The possible reason can be attributed to an improvement of the oxidizability of CuO*_x_* species during the high-temperature treatment in the first run.

**Figure 10:**
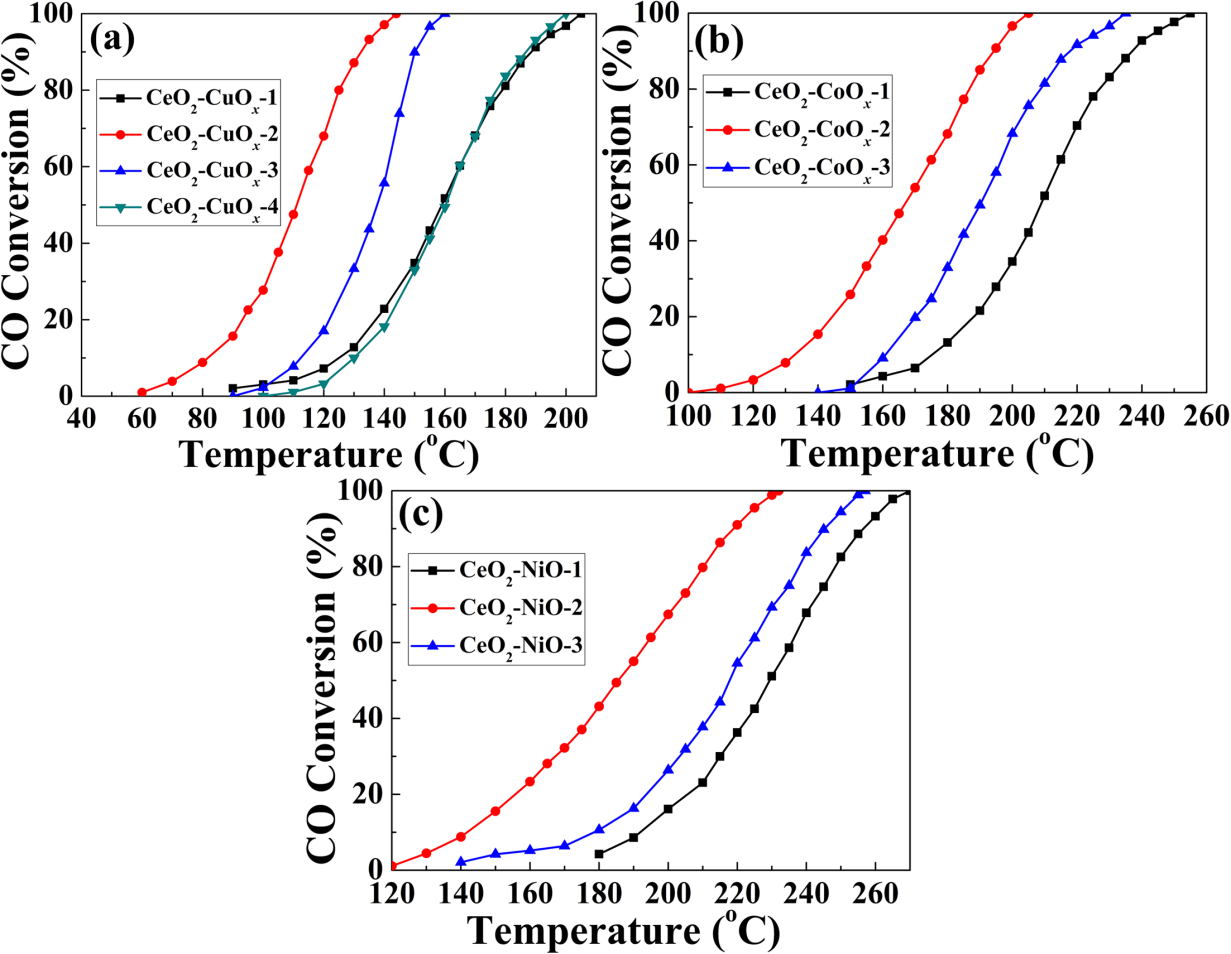
CO conversion as a function of temperature for CeO_2_–MO*_x_* composite yolk–shell nanospheres obtained with different initial concentrations of M(CH_3_COO)_2_·*x*H_2_O: (a) CeO_2_–CuO*_x_*; (b) CeO_2_–CoO*_x_* and (c) CeO_2_–NiO.

The initial concentration of M(CH_3_COO)_2_·*x*H_2_O in the synthesis process played a significant role in catalytic performance for CO oxidation. By simply varying the amount of M(CH_3_COO)_2_·*x*H_2_O, a series of composite nanospheres was prepared to investigate the effects on the catalytic activity (the detailed synthesis is given in Experimental section). The CO conversion curves are shown in Figure 10. Each sample exhibits enhanced catalytic performance in comparison with naked CeO_2_. For CeO_2_–CuO*_x_*, the corresponding samples were denoted as CeO_2_–CuO*_x_*–1, CeO_2_–CuO*_x_*–2, CeO_2_–CuO*_x_*–3 and CeO_2_–CuO*_x_*–4 obtained by addition of 0.01, 0.02, 0,04 and 0.08 mmol of Cu(CH_3_COO)_2_·H_2_O, respectively. CeO_2_–CuO*_x_*–1 sample exhibited a *T*_100_ value of 235 °C (Figure 10a). The value of *T*_100_ of CeO_2_–CuO*_x_*–2 sample sharply decreased to 145 °C. Upon a further increase of the initial concentration of Cu(CH_3_COO)_2_·H_2_O, the catalytic activity of the as-obtained samples deteriorated, with *T*_100_ values of 160 °C for CeO_2_–CuO*_x_*–3 and 230 °C for CeO_2_–CuO*_x_*–4. Among the series of CeO_2_–CoO*_x_* and CeO_2_–NiO samples, the catalytic performance exhibited a similar trend (Figure 10b,c). CeO_2_–CoO*_x_*–2 and CeO_2_−NiO−2 showed the optimal catalytic performance, and *T*_100_ values of 203 and 235 °C for CeO_2_–CoO*_x_*–2 and CeO_2_–NiO–2, both of which were obtained with 0.04 mmol of Co(CH_3_COO)_2_·4H_2_O and Ni(CH_3_COO)_2_·4H_2_O, respectively. The results indicate that the introduction of a proper amount of MO*_x_* species results in significantly higher catalytic activity of the CeO_2_–MO_x_ samples. However, an excessive amount of MO*_x_* introduced into CeO_2_ may block the pore channels generated during the heating process and cover the active sites of CeO_2_, leading to a decline of catalytic activity.

In principle, the catalytic process mainly involves the adsorption and desorption of gas molecules at the interface or on the surface during CO oxidation over metal-oxide catalysts. According to previously reported literature, the possible CO oxidation reaction mechanism over the ceria-based mixed oxides could be tentatively proposed as follows: CO + M–[O]–Ce–□_vac_ + 1/2 O_2_ → [CO–M–O–Ce–O]* → CO_2_ (g) + M–[O]–Ce–□_vac_, where M–[O]–Ce denotes a metal species incorporated into CeO_2_, and □_vac_ denotes an oxygen vacancy [39]. Essentially, the interaction between CeO_2_ and MO*_x_* is due to the lengthening and weakening of the M–O bond by sharing oxygen at the interface [40]. Therefore, the molecular oxygen activation and extraction preferentially takes place at the interface site between MO*_x_* and CeO_2_. During CO oxidation over the CeO_2_–MO*_x_* catalyst, MO*_x_* provides chemisorption sites for CO molecules, while CeO_2_ traps oxygen molecules with oxygen vacancies and increases the concentration of surface oxygen due to its excellent oxygen storage capacity. In the CO oxidation process, oxygen atoms will transfer from molecular oxygen to the MO*_x_* clusters through the CeO_2_, which is in close contact with MO*_x_* at the two-phase interface, remarkably promoting oxygen mobility on the surface of CeO_2_–MO*_x_* composite and effectively facilitating molecular oxygen activation [41–42].

The CeO_2_–CuO*_x_* catalyst exhibited much better activity than the other samples, indicating that the catalytic activity may be related with the intrinsic property of the doped metal and the metal-oxide interactions. For MO*_x_*-decorated CeO_2_ nanostructures, the enhanced redox properties at the interface sites play a key role in the superior catalytic efficiency in the oxidation reaction [33]. The charge balance of redox couples of Cu^2+^/Cu^+^ and Ce^4+^/Ce^3+^ (Ce^3+^ + Cu^2+^ ↔ Ce^4+^ + Cu^+^) and the lowering of redox potentials of Cu species interacting with CeO_2_ support could be responsible for the improved catalytic performance of the CeO_2_–CuO*_x_* catalyst [43]. For Ce–Co and Ce–Ni binary oxides, Co^3+^/Co^2+^ and Ni^3+^/Ni^2+^ charge pairs will also be balanced with Ce^4+^/Ce^3+^ pair at the two-phase interface [44]. Nevertheless, since the redox potentials follow the sequence of Cu^2+^/Cu^+^ << Co^3+^/Co^2+^ ≈ Ni^3+^/Ni^2+^, Cu will be more effective than Co or Ni to achieve a charge balance with Ce^4+^/Ce^3+^, leading to more pronounced catalytic activity for CO oxidation [39,44]. In addition, the adsorption capacity for CO molecules is another important factor that influences the catalytic activities. Previous studies have shown that Ni and Co cations are less efficient for CO chemisorption than Cu cations, resulting in inferior CO oxidation activity [44–45]. As previously reported, the catalytic activities of 5% doped M–CeO_2_ (M = Cu, Co and Ni) catalyst were evaluated, and the order of reaction rates in CO oxidation were Cu > Co > Ni > undoped [39,43], which is in accordance with the results of our present study.

## Conclusion

In summary, uniform CeO_2_–MO*_x_* (M = Cu, Co, Ni) composite yolk–shell nanospheres have been successfully prepared by a general approach, consisting of the calcination of solid Ce-CPCSs precursor to produce CeO_2_ yolk–shell nanospheres and the subsequent solvothermal treatment with M(CH_3_COO)_2_. Preliminary catalytic experiments indicate that the CeO_2_–MO*_x_* composite nanospheres exhibited excellent catalytic activity toward CO oxidation. Additionally, cycling test confirms an excellent catalytic stability and durability during the CO oxidation process. The initial concentration of M(CH_3_COO)_2_·*x*H_2_O in the synthesis process played a significant role in catalytic performance. The catalytic activity of the CeO_2_–CuO*_x_*-2 sample is comparable to the traditional noble-metal–CeO_2_ system, yielding complete CO conversion at a relatively low temperature of 145 °C. A greatly enhanced performance of the composites in CO oxidation can be attributed to the incorporation of highly-dispersed MO*_x_* onto the CeO_2_ surface and the strong synergistic interaction between MO*_x_* species and CeO_2_. Additionally, the CeO_2_–CuO*_x_* catalyst is more active than the CeO_2_–CoO*_x_* and CeO_2_–NiO catalysts for the CO oxidation, suggesting that the catalytic activity is mainly related to the intrinsic property of the doped metal and the metal-oxide interactions. It may be tentatively explained that the Co and Ni absorb less CO molecules less efficiently than Cu and are less effective in balancing charges with the Ce^4+^/Ce^3+^ pair. This synthesis approach could be further applied to create other CeO_2_-based composite oxides with various nanostructures for a broad range of technical applications.

## Experimental

### Materials

Cerium(III) nitrate hexahydrate (Ce(NO_3_)_3_·6H_2_O), concentrated nitric acid (HNO_3_, 68%), diethylene glycol (DEG), acetone, copper(II) acetate monohydrate (Cu(CH_3_COO)_2_·H_2_O), nickel(II) acetate tetrahydrate (Ni(CH_3_COO)_2_·4H_2_O), cobalt(II) acetate tetrahydrate (Co(CH_3_COO)_2_·4H_2_O) and absolute ethanol were purchased from Sinopharm Chemical Reagent Co. Ltd.. All the chemical reagents are analytically pure and used as received without further purification.

### Synthesis of CeO_2_ yolk–shell nanospheres

The Ce-CPCSs were synthesized according to [27]. In a typical procedure, 0.109 g of Ce(NO_3_)_3_·6H_2_O was dissolved in 5 mL of diethylene glycol under vigorous stirring. Subsequently, 2 mL of concentrated nitric acid and 35 mL of acetone were added to the above solution in sequence, and stirring was continued for another 30 min to form a clear solution. The resultant mixture was placed to a 50 mL Teflon-lined stainless steel autoclave and maintained at 100 °C for 10 h. After cooling to room temperature, the Ce-CPCSs were collected by centrifugation, washed with ethanol three times and oven-dried at 60 °C. The CeO_2_ yolk–shell nanospheres were completed by thermal decomposition of the Ce-CPCSs at 500 °C for 4 h in air with a heating rate of 8 °C/min.

### Synthesis of CeO_2_–MO*_x_* composite yolk–shell nanospheres

In a typical process, 0.04 mmol of M(CH_3_COO)_2_·*x*H_2_O (M = Cu, Co, Ni) was dissolved in 16 mL of absolute ethanol under vigorous stirring. Then 50 mg of the as-prepared CeO_2_ was dispersed into the above clear solutions under ultrasonification. Subsequently, the resultant homogeneous slurry was placed in a 25 mL Teflon-lined stainless steel autoclave and maintained at 120 °C for 12 h. Finally, the products were harvested by centrifugation and thoroughly washed with deionized water three times before being dried at 80 °C in an electric oven.

For comparison, a series of CeO_2_–MO_x_ samples were synthesized by addition of initial concentrations of M(CH_3_COO)_2_·*x*H_2_O (M = Cu, Co, Ni), while other conditions were kept unchanged. For CeO_2_–CuO*_x_* samples, 0.01, 0.02, 0.04 and 0.08 mmol of Cu(CH_3_COO)_2_·H_2_O were introduced, the corresponding samples were named CeO_2_–CuO*_x_*–1, CeO_2_–CuO*_x_*–2, CeO_2_–CuO*_x_*–3 and CeO_2_–CuO*_x_*–4, respectively. For CeO_2_–CoO*_x_* and CeO_2_–NiO samples, 0.028, 0.04 and 0.052 mmol M′(CH_3_COO)_2_·4H_2_O (M′ = Co, Ni) was added, the corresponding products were denoted CeO_2_–M′O*_x_*–1, CeO_2_–M′O*_x_*–2 and CeO_2_–M′O*_x_*–3, respectively.

### Characterization

Crystallographic phases and purity were investigated by X-ray diffraction (XRD) on a Bruker D8-Advance powder X-ray diffractometer with Cu Kα radiation (λ = 0.15418 nm). The morphologies and structures were examined by scanning electron microscopy (SEM) using a FEI Quanta^TM^ 250 and transmission electron microscopy (TEM) using a FEI Tecnai G2 F20, equipped with an energy dispersive X-ray spectrometer (EDX) for elemental mapping. Raman spectra were carried out using a Bruker Senterra Raman spectrometer with an excitation laser of 532 nm. Surface analysis was obtained by an X-ray photoelectron spectroscopy (XPS, Thermo ESCALAB 250Xi) with Al Kα radiation. All binding energies were corrected for surface charging by use of the C 1s peak (284.8 eV) of adventitious carbon as reference. The M contents in CeO_2_–MO*_x_* samples were analyzed by inductively coupled plasma mass spectrometry (ICP-MS, Agilent NWR 213-7900). Hydrogen temperature programmed reduction (H_2_-TPR) experiment was performed on a PCA-1200 instrument, equipped with a thermal conductivity detector (TCD) to detect H_2_ consumption. Typically, 50 mg of the sample was heated (10 °C/min) from room temperature to 700 °C in a 5 vol % H_2_/Ar gaseous mixture with a flow rate of 30 mL/min.

### Catalytic tests

The activity measurements were carried out in a continuous flow fixed-bed microreactor at atmospheric pressure. In a typical experiment, 50 mg of catalyst with 500 mg silica sand was loaded into a stainless steel tube. A gas mixture of CO/O_2_/N_2_ (1:10:89) with a total flow rate of 50 mL/min flowed through the reactor, equivalent to a weight hourly space velocity (WHSV) of 60000 mL·g^−1^·h^−1^. The composition of the gas exiting from the reactor was monitored with an online infrared gas analyzer (Gasboard-3100, China Wuhan Cubic Co.) that can simultaneously detect CO, CO_2_ and O_2_. The CO conversion ratio was calculated based on the CO consumption and CO_2_ formation.
